# Serum levels of adiponectin and leptin as biomarkers of proteinuria in lupus nephritis

**DOI:** 10.1371/journal.pone.0184056

**Published:** 2017-09-12

**Authors:** Valeria Diaz-Rizo, David Bonilla-Lara, Laura Gonzalez-Lopez, Dalia Sanchez-Mosco, Nicte S. Fajardo-Robledo, Edsaul E. Perez-Guerrero, N. Alejandra Rodriguez-Jimenez, A. Miriam Saldaña-Cruz, M. Luisa Vazquez-Villegas, Eduardo Gomez-Bañuelos, Monica Vazquez-Del Mercado, E. German Cardona-Muñoz, David Cardona-Muller, Xochitl Trujillo, Miguel Huerta, Mario Salazar-Paramo, Jorge I. Gamez-Nava

**Affiliations:** 1 Unidad de Investigacion Biomedica 02, Hospital de Especialidades, Centro Medico Nacional de Occidente (CMNO), Instituto Mexicano del Seguro Social (IMSS), Guadalajara, Jalisco, Mexico; 2 Programa de Posgrado de Ciencias Medicas, Universidad de Colima, Colima, Mexico; 3 Programa de Posgrado en Farmacologia, CUCS, Universidad de Guadalajara, Guadalajara, Jalisco, Mexico; 4 Departamento de Medicina Interna-Reumatologia, Hospital General Regional 110 (HGR 110), IMSS, Guadalajara, Jalisco, Mexico; 5 Laboratorio de Investigación y Desarrollo Farmacéutico, Centro Universitario de Ciencias Exactas e Ingenierías, Universidad de Guadalajara, Guadalajara, Jalisco, México; 6 Departamento de Farmacobiologia, Centro Universitario de Ciencias Exactas e Ingenierías, Universidad de Guadalajara, Guadalajara, Jalisco, México; 7 Departamento de Ciencias Biomedicas, Centro Universitario Tonala, Universidad de Guadalajara, Guadalajara, Jalisco, Mexico; 8 Departamento de Epidemiologia, Unidad Medica Familiar 4 y 8, IMSS, Guadalajara, Jalisco, Mexico; 9 Programa de Posgrado en Salud Publica, CUCS, Universidad de Guadalajara, Guadalajara, Jalisco, Mexico; 10 Instituto de Investigacion en Reumatologia y Sistema Musculoesqueletico, Centro Universitario de Ciencias de la Salud, Universidad de Guadalajara, Guadalajara, Jalisco, Mexico; 11 Servicio de Reumatologia, Division de Medicina Interna OPD, Hospital Civil Juan I. Menchaca, Guadalajara, Jalisco, Mexico; 12 Instituto de Terapeutica Experimental y Clinica, Departamento de Fisiologia, CUCS, Universidad de Guadalajara, Guadalajara, Jalisco, Mexico; 13 Division de Investigacion en Salud, Unidad Medica de Alta Especialidad, Hospital de Especialidades, Centro Medico Nacional de Occidente (CMNO), Instituto Mexicano del Seguro Social (IMSS), Guadalajara, Jalisco, Mexico; SERGAS and IDIS, SPAIN

## Abstract

**Introduction:**

There are controversial results about the role of serum leptin and adiponectin levels as biomarkers of the severity of proteinuria in lupus nephritis.

**Objective:**

The aim of this study was to evaluate the relationship between serum leptin and adiponectin levels with severity of proteinuria secondary to lupus nephritis (LN).

**Methods:**

In a cross-sectional study, 103 women with systemic lupus erythematosus (SLE) were evaluated for kidney involvement. We compared 30 SLE patients with LN, all of them with proteinuria, versus 73 SLE patients without renal involvement (no LN). A comprehensive set of clinical and laboratory variables was assessed, including serum levels of leptin and adiponectin by ELISA. Multivariate analyses were used to adjust for potential confounders associated with proteinuria in LN.

**Results:**

We found higher adiponectin levels in the LN group compared with the no LN group (20.4 ± 10.3 vs 15.6 ± 7.8 μg/mL; p = 0.02), whereas no differences were observed in leptin levels (33.3 ± 31.4 vs 22.5 ± 25.5 ng/mL; p = 0.07). Severity of proteinuria correlated with an increase in adiponectin levels (r = 0.31; p = 0.001), but no correlation was observed with leptin. Adiponectin levels were not related to anti-dsDNA or anti-nucleosome antibodies. In the logistic regression, adiponectin levels were associated with a high risk of proteinuria in SLE (OR = 1.06; 95% CI 1.01–1.12; p = 0.02). Instead, leptin was not associated with LN.

**Conclusion:**

These findings indicate that adiponectin levels are useful markers associated with proteinuria in LN. Further longitudinal studies are required to identify if these levels are predictive of renal relapse.

## Introduction

Lupus nephritis (LN) comprises one of the most severe, life-threatening complications in systemic lupus erythematosus (SLE) [1]. Patients with SLE have an overall 10 years survival rate of 92%, in comparison to 88% in patients with renal involvement [2,3]. Proteinuria is one of the cornerstone features to identify renal disease activity and is a useful biomarker to modify treatment strategies in LN. In a retrospective study, some authors identified that high levels of proteinuria are associated with lower probability of full recovery of renal function in LN during follow-up [4]. In consequence, a number of serological biomarkers related with LN and proteinuria have been tested. Abnormalities in adipokine levels have been proposed to be co-participants in the inflammatory processes observed in SLE, with a potential role in some inflammatory components including T-cell regulation [5,6]. Furthermore, increased serum levels of different adipokines mainly leptin and adiponectin have been observed in several rheumatic autoimmune diseases such as rheumatoid arthritis (RA) [7]. Leptin is probably the main adipokine evaluated on patients with rheumatic autoimmune diseases. This adipokine has an active role in the modulation of innate and adaptive immunity. Leptin levels are increased in osteoarthritis and RA [8]. Different studies have demonstrated in human and murine chondrocytes a catabolic role of leptin mediated by an activation of type 2 nitric oxide synthase (NOS2) triggering metalloproteinases activation (MMPs), and chondrocyte apoptosis [8]. In some chronic inflammatory diseases and animal models, this hormone-like molecule exhibits pro-inflammatory characteristics, including a decrease on T-cells apoptosis and enhancement of the activation of T-helper 1 cells (TH1), leading to an increase in the synthesis of pro-inflammatory cytokines and monocytes activation [9]. Effects of altered leptin levels are complex in SLE; some authors have observed an association between high levels of leptin, with an increase of C-reactive protein levels (CRP) and disease activity in SLE [10,11]. Instead, other authors, such as Hutcheson et al., did not observe a correlation between serum leptin levels and disease activity assessed by the SLEDAI [12]. On the other hand, adiponectin seems to play a more complex role in chronic inflammatory diseases because it also exhibits anti-inflammatory effects. These interesting aspects of adiponectin have been reviewed extensively by Scotece et al. [8]. Adiponectin has pro-inflammatory effects producing inhibition of phagocytic activity on macrophages, decreasing tumor necrosis factor-alpha (TNF-α) and interleukin (IL)-6 production [13]. In contrast, on other cells such as human chondrocytes adiponectin has a pro-inflammatory role inducing increased levels of NOS2, IL-6, metalloproteinases 3 and 9 and also enhances monocyte chemoattractant protein-1 (MCP-1) production, whereas in synovial fibroblasts adiponectin induces IL-6, MMP-1 and IL-8 production, in addition high levels of adiponectin have been correlated with the severity and joint destruction in RA patients [8]. Moreover, Frommer et al, have described on synovial fibroblasts derived from RA patients a differential induction of pro-inflammatory cytokines and MMP regulated depending on individual adiponectin isoforms [14]. On the other side, the role of adiponectin in renal involvement of glomerulonephritis including rheumatic diseases has not been consistently demonstrated and discordant results about the relation of adiponectin levels and proteinuria have been observed in different diseases [15–19]. Hayakawa et al., observed a positive correlation between plasma adiponectin levels and proteinuria in patients with glomerulonephritis, although they did not make reference to SLE [20]. Some studies have explored if there are increases in adipokine levels in SLE [12,17,18,20–22]. Hutcheson et al., observed that leptin and adiponectin increased in LN versus controls [12]. Rovin et al., identified increased plasma levels of adiponectin in SLE with renal involvement in comparison with SLE without renal flare, but these levels were not significant in the multivariate analysis and the association of adiponectin levels and renal involvement was found only in the univariate analysis, and did not remain after adjusting for confounders [18]. Other authors have observed an increase in urinary adiponectin in LN correlating with active renal damage, although no assessment of leptin was included in these studies [17,22]. These discordances could be partially explained because most of the studies had a lack of controlling confounders. In consequence, the role of these adipokines as biomarkers of LN with proteinuria should be considered in multivariate models. Therefore, our objective was to identify the value of leptin and adiponectin levels as biomarkers associated with proteinuria in LN after adjusting for potential confounders using robust multivariate models.

## Materials and methods

We conducted a cross-sectional study that included patients with SLE attending an outpatient rheumatology clinic. The patients were females, were ≥18 years of age, were born in Mexico, and met the 1982 American College of Rheumatology criteria for SLE [23]. We excluded patients with any of the following conditions: i. active infections (particularly infection of urinary tract), ii. overlap syndrome, iii. diabetes mellitus, iv. thyroid disease, v. pregnancy or breastfeeding, vi. cancer, vii. history of blood transfusion in the last six months, and other causes of proteinuria not related with LN. We included clinically healthy females as the comparison group, who were seen at a department of preventive medicine clinic located at the same hospital as the SLE patients. This comparison group was matched with patients in a similar age range.

### Clinical assessment in SLE

A structured assessment for SLE was performed by trained researchers, including the following clinical variables: age, SLE disease duration, antecedents of previous episodes of LN, other organ involvement, comorbidities, and treatment. We included the assessment of some epidemiological risk factors, including the variable of alcohol abuse. This variable was operatively defined using the World Health Organization (WHO) criteria described by the Expert Committee on Drug Dependence and by the Diagnostic and Statistical Manual of Mental Disorders (DSM IV-TR), which defines alcohol abuse as an inadequate pattern of drinking, leading to failure to fulfill their job, home, or school obligations and putting their legal or social stability at risk [24].

Fasting blood glucose and serum creatinine were quantified in SLE and controls. Urinalysis was carried out to assess the presence of casts, erythrocyturia (>5 erythrocytes/field), red blood cells (RBC), and white blood cell (WBC) counts. 24-hour urine was collected to estimate proteinuria (g/day), creatinine clearance (mL/min) and the estimated glomerular filtration rate (eGFR, mL/min/m^2^ body surface). Disease activity was evaluated with the SLE activity index (SLEDAI) [25]. Renal disease activity was assessed with renal-SLEDAI (rSLEDAI), which included proteinuria, urine casts, erythrocyturia, and pyuria [25]. Organ damage was identified using the systemic lupus international collaborating clinics/ACR damage index (SLICC/ACR) [26]. Other assessments included waist and hip circumferences and body mass index (BMI), calculated as the weight in kilograms divided by the square of height in meters (kg/m^2^), as stated in the international classification of adults according to the WHO. BMI index was operationally classified according to the international classification of adult overweight and obesity in three categories: normal weight (18.5 to 24.9 kg/m^2^), overweight (25.0 to 29.9 kg/m^2^), and obese (≥30.0 kg/m^2^). Body fat mass (BFM) was measured by bioelectrical impedance (TANITA InnerScan BC-533; TANITA, Tokyo, Japan).

### Comparison groups

Patients with SLE were classified into two groups: a) Lupus nephritis and proteinuria (>0.5 g/day) (LN) and b) SLE without nephritis (no NL) (proteinuria ≤0.5 g/day) [25]. Healthy controls were included as a comparison group for normal values.

### Quantification of leptin and adiponectin levels

All of the patients with SLE and controls were assessed for leptin and adiponectin quantification in serum obtained with >12-h fasting. Serum leptin and adiponectin concentrations were measured by ELISA (Quantikine Human Leptin or Adiponectin Immunoassay; R&D Systems, Germany). Leptin sensitivity was <7.8 pg/mL, and adiponectin sensitivity was 0.246 ng/mL. All procedures for the measurement of adipokines were conducted by researchers that were blind to the patients’ clinical characteristics. Serum leptin/BMI and adiponectin/BMI indices were obtained as surrogate measures of these two adipokines adjusted by BMI, a confounder related with the levels of these molecules. Leptin/BMI ratio has been used by Lee et al., [27] whereas Cohen et al., [28] used the adiponectin/BMI ratio to control the influence of BMI on these adipokines levels.

### Statistical analysis

Quantitative variables were described as means ± standard deviations (SD), and qualitative variables were described as frequencies (%). Student t-test was used to compare means of quantitative variables between groups. A chi-square test was performed to compare proportions of categorical variables. Correlation analyses (Pearson tests) were computed to examine the strength of association between adipokine concentration and quantitative variables, including proteinuria, eGFR, and rSLEDAI. A logistic regression analysis was performed in order to assess the influence of these adipokines on the presence of proteinuria in SLE adjusted by potential confounders. In order to build the final model, the dependent variable was the presence of proteinuria (>0.5 g/day) and the covariates included in the models were those variables that had a p-value <0.20 in the univariate analysis or those variables with biological plausibility to be associated with proteinuria. We used Enter method to test the significant association of the covariates introduced in the models and the Forward stepwise method to present the results of the adjusted model. All the models were adjusted by age and disease duration of SLE. In this final model, we present adjusted odds ratios (ORs) and their 95% confidence intervals (95% CI) meaning the risk that confers these variables for proteinuria secondary to LN. Variables that in the adjusted analysis had ORs higher than 1 were considered as risk factors for proteinuria, whereas variables that obtained ORs lower than 1 in the adjusted analysis were considered as protective factors for this feature of renal involvement in SLE only if the range of their 95% CI did not include the value of 1. Statistical significance was set at a p-value <0.05. SPSS (version 21; SPSS, Inc., Chicago, IL, USA) was used for the statistical analysis.

### Ethics

This study protocol was revised and approved by the Research and Ethics Board of the hospital (name in Spanish of the approving committee: “Comite Local de Investigación en Salud Núm 1301”). Approval ID: R-2010-1301-35. Each procedure conducted during the study was performed following the guidelines of the Declaration of Helsinki. All participants signed informed consent prior to study onset.

## Results

One hundred three females with SLE and 83 female controls were enrolled in this study. The mean age of patients with SLE was 43 years, with a mean SLEDAI score of 3. All patients were receiving glucocorticoids. Selected demographic and clinical characteristics of patients with SLE and control groups are shown in Table 1.

**Table 1:** Selected clinical characteristics of patients in the study and of the control group.

Characteristics	SLE	Controls	
	*n* = 103	*n* = 83	*p*
Age, years (mean SD)	42.6 ± 11.3	44.6 ± 10.0	0.24
Gender (feminine), *n* (%)	103 (100.0)	83 (100.0)	-
Weight, kg (mean ± SD)	68.6 ± 13.4	68.9 ± 12.9	0.90
BMI, kg/m^2^ (mean ± SD)	27.3 ± 4.9	27.5 ± 4.9	0.85
Normal, 18.5–24.9 kg/m^2^; *n* (%)	33 (32.0)	29 (34.0)	0.90
Overweight, 25.0–29.9 kg/m^2^; *n* (%)	37 (35.9)	31 (37.3)	
Obesity, ≥30.0 kg/m^2^; *n* (%)	26 (25.2)	22 (26.5)	
Leptin (ng/mL)	25.7 ± 27.6	19.7 ± 19.0	0.09
Leptin/BMI ratio	0.9 ± 1.0	0.7 ± 0.6	0.08
Adiponectin (μg/mL)	17.0 ± 8.9	12.0 ± 6.5	<0.001
Adiponectin/BMI ratio	0.7 ± 0.4	0.5 ± 0.3	<0.001
Disease duration, years (mean ± SD)	9.1 ± 6.3	-	-
Active SLE (score ≥3), *n* (%)	39 (37.9)	-	-
SLEDAI score (mean ± SD)	2.5 ± 3.1	-	-
rSLEDAI, *n* (%)	30 (29.1)	-	-
SLICC/ACR score (mean ± SD)	0.8 ± 0.9	-	-
Corticosteroid use, *n* (%)	103 (100.0)	-	-
Corticosteroid doses, mg/day (mean ± SD)	14.1 ± 15.3	-	-

Data is shown in means ± Standard Deviations (SD). Qualitative variables are expressed in frequencies (%). SLE: Systemic Lupus Erythematosus; BMI: Body Mass Index; SLEDAI: SLE Disease Activity Index; rSLEDAI: renal-SLEDAI; Systemic Lupus International Collaborative Clinics/American College of Rheumatology (SLICC/ACR) Damage Index.

Adiponectin and adiponectin/BMI ratio were both increased in SLE patients in comparison to controls (p<0.001), whereas a non-significant trend to higher leptin levels (p = 0.09) and leptin/BMI ratio (p = 0.08) was observed in SLE patients. Proteinuria was identified in 30.9% of SLE patients. All patients were classified with renal activity according to rSLEDAI-presented proteinuria (˃0.5 g/day). Therefore, patients with SLE were classified into two groups: 30 with proteinuria (LN) and 73 without LN, defined as no lupus nephritis (non-LN) (Table 2).

**Table 2:** Comparison of clinical characteristics, leptin, and adiponectin levels between patients with systemic lupus erythematosus (SLE) with proteinuria vs. those without lupus nephritis (LN).

Variable	SLE		
	LN	No LN	
	*n* = 30	*n* = 73	*p*
Age (years)	39.1 ± 10.7	44.0 ± 11.2	0.04
Weight (kg)	68.9 ±14.8	68.5 ± 12.9	0.91
Body mass index (BMI) (kg/m^2^)	26.9 ± 5.2	27.5 ± 4.8	0.58
Normal, 18.5–24.9; *n* (%)	10 (33.3)	23 (31.5)	0.95
Overweight, 25.0–29.9; *n* (%)	10 (33.3)	27 (37.0)	
Obesity, ≥30.0; *n* (%)	10 (33.3)	22 (30.1)	
Body fat mass (%)	34.9 ± 8.0	36.3 ± 4.1	0.45
Alcohol abuse, *n* (%)	1 (3.3)	6 (8.2)	0.77
Smoking, *n* (%)	3 (10.0)	2 (2.7)	0.15
Disease duration (years)	7.3 ± 5.1	9.9 ± 6.7	0.04
SLEDAI score	5.8 ± 2.9	1.1 ± 1.9	<0.001
Active SLE (>3), *n* (%)	30 (100.0)	10 (13.7)	<0.001
SLICC/ACR score	1.4 ± 0.8	0.6 ± 0.8	<0.001
Leptin (ng/mL)	33.3 ± 31.4	22.5 ± 25.5	0.07
Leptin/BMI ratio	1.2 ± 1.0	0.8 ± 0.9	0.07
Adiponectin (μg/mL)	20.4 10.3	15.6 ± 7.8	0.02
Adiponectin/BMI ratio	0.8 ± 0.5	0.60 ± 0.4	0.02
Anti-dsDNA antibody (IU/mL)	212.1 ± 243.3	175.9 ± 245.7	0.54
Anti-nucleosome antibody (RU/mL)	51.2 ± 73.3	15.5 ± 36.9	0.03
Corticosteroids mean doses (mg/day)	24.0 ± 22.0	10.0 ± 8.9	0.002
Azathioprine users, *n* (%)	16 (59.3)	38 (52.1)	0.65
Cyclophosphamide users, *n* (%)	3 (11.1)	4 (5.6)	0.39
Mycophenolate Mofetil users, *n* (%)	12 (44.4)	18 (24.7)	0.08

LN: Lupus Nephritis; No NL: No Lupus Nephritis. Proteinuria was considered as ≥0.5 g/day. The alcohol abuse was considerate as 'persistent or sporadic excessive alcohol use inconsistent with or unrelated to acceptable medical practice' (WHO, 1969). Quantitative variables are expressed as mean ± Standard Deviations (SD), and qualitative variables are expressed as frequencies (%). Student’s *t*-test was used for comparisons between means, and Chi-squared test was used for comparison between proportions (Fisher exacted if appropriated).

In the comparison between groups, patients with LN were younger (p = 0.04). Adiponectin levels were higher in the proteinuria group (LN) compared with patients without renal involvement (20.4 vs. 15.6 μg/mL, respectively; p = 0.02). Also, the adiponectin/BMI ratio was higher in the LN group compared to SLE patients without renal involvement (0.8 vs. 0.6; p = 0.02). A non-significant trend for higher leptin levels was observed in the LN group (p = 0.07). Similarly, the leptin/BMI ratio in LN patients was higher but did not reach statistical significance (p = 0.07). Higher levels of antinucleosome antibodies were observed in LN patients compared with patients without LN (51.2 vs. 15.5 relative units/milliliter (RU/mL); p = 0.03) (Table 2). On the other hand, leptin levels were correlated with BMI (r = 0.35; p <0.001), BFM (r = 0.24, p = 0.03), and corticosteroids doses (r = 0.22; p = 0.03). A significant correlation between serum adiponectin levels and high levels of proteinuria (r = 0.31; p = 0.001), serum creatinine (r = 0.2; p = 0.05), corticosteroid doses (r = 0.38; p <0.001), SLEDAI (r = 0.20; p = 0.05), and rSLEDAI (r = 0.23; p = 0.02) was observed, although no correlation was observed between adiponectin levels and eGFR (r = -0.04, p = 0.71). An increase in adiponectin levels had an inverse correlation with BMI (r = –0.25; p = 0.01) and BFM (r = –0.35; p = 0.001). There was no correlation between serum leptin levels and proteinuria (r = 0.004; p = 0.97) neither with eGFR (r = -0.16; p = 0.10). There was a borderline negative correlation between the leptin/BMI ratio and eGFR (r = –0.20; p = 0.05) or (r = -0.004; p = 0.97). There was a positive significant correlation between rSLEDAI score and leptin levels (r = 0.22; p = 0.03), as well as with leptin/BMI ratio (r = 0.26; p = 0.009) (Table 3). We also assessed the correlation between proteinuria with other variables. Proteinuria correlated with SLEDAI score (r = 0.44; p <0.001), anti-double-stranded DNA (anti-dsDNA) titers (r = 0.37; p = 0.001), corticosteroid doses (r = 0.44; p <0.001), and the adiponectin/BMI ratio (r = 0.28; p = 0.004). A negative correlation between proteinuria and age was observed (r = –0.31; p = 0.002). Nonetheless, no correlation between proteinuria and complement C3 (r = –0.12; p = 0.28) and C4 (r = –0.07; p = 0.55) was observed.

**Table 3:** Clinical and serological features correlated with leptin, leptin/BMI ratio, adiponectin, and adiponectin/BMI ratio.

Variables	Leptin		Leptin/BMI ratio		Adiponectin		Adiponectin/BMI ratio	
	*r*	*p*	*r*	*p*	*r*	*p*	*r*	*p*
Age (years)	- 0.05	0.65	-0.06	0.54	-0.13	0.21	-0.18	0.07
Weight (kg)	0.37	<0.001	0.22	0.03	-0.25	0.01	-0.47	<0.001
BMI (kg/m^2^)	0.35	<0.001	0.19	0.05	-0.25	0.01	-0.49	<0.001
Body fat mass %	0.24	0.03	0.14	0.19	-0.35	0.001	-0.52	<0.001
SLEDAI score	0.14	0.15	0.18	0.07	0.20	0.05	0.18	0.16
rSLEDAI score	0.22	0.03	0.26	0.009	0.23	0.02	0.21	0.04
SLICC/ACR score	-0.01	0.90	0.002	0.99	0.11	0.27	0.09	0.38
Disease duration	-0.07	0.47	-0.11	0.27	-0.07	0.49	-0.13	0.19
Proteinuria (g/day)	0.004	0.97	-0.004	0.97	0.31	0.001	0.28	0.004
Creatinine clearance (mL/min)	-0.003	0.98	-0.008	0.94	-0.1	0.33	-0.10	0.31
Estimated glomerular filtration rate (eGFR) (mL/min/m^2^)	-0.16	0.10	-0.20	0.05	-0.04	0.71	-0.03	0.74
Serum creatinine (mg)	0.12	0.24	0.17	0.10	0.2	0.05	0.21	0.04
Corticosteroid doses (mg/day)	0.22	0.03	0.22	0.02	0.38	<0.001	0.33	0.001
Anti-dsDNA (IU/mL)	-0.12	0.31	-0.13	0.27	0.15	0.19	0.15	0.18
Anti-nucleosome (RU/mL)	0.02	0.90	0.009	0.94	0.11	0.38	0.06	0.61
C3	-0.03	0.81	-0.05	0.66	-0.12	0.25	-0.17	0.12
C4	-0.05	0.65	-0.08	0.44	-0.17	0.12	-0.23	0.03

BMI: Body Mass Index. SLEDAI: SLE Disease Activity Index; rSLEDAI: renal-SLEDAI; SLICC/ACR Systemic Lupus International Collaborative Clinics/American College of Rheumatology Damage Index. Pearson (*r*) was used to compute the correlations between variables.

In data that are not depicted in tables leptin, adiponectin levels, leptin/BMI and adiponectin/BMI did not correlate with serum levels of anti-dsDNA or anti-nucleosome antibodies.

Table 4 presented a logistic regression analysis evaluating factors associated with the presence of proteinuria in LN (>0.5 g/day), where the covariates were age, BMI, disease duration, SLEDAI, corticosteroid doses, leptin, and adiponectin. In the forward stepwise method, we observed that higher levels of adiponectin increased the risk of proteinuria in SLE patients (OR = 1.06, 95% CI 1.01–1.12; p = 0.02). Instead, leptin levels were not associated with proteinuria in LN.

**Table 4:** Logistic regression analysis evaluating factors associated with presence of proteinuria in systemic lupus erythematosus (SLE).

	*Enter* Method			*Forward Stepwise* Method		
Predictor criterion	OR	95% CI	*p*	OR	95% CI	*p*
Age, years	0.97	0.93–1.01	0.13	0.96	0.92–1.00	0.05
BMI (kg/m^2^)	0.99	0.88–1.10	0.79	Not in the model	–	–
Disease duration, years	0.95	0.88–1.04	0.29	Not in the model	–	–
Leptin, ng/mL	1.02	1.0–1.03	0.13	Not in the model	–	–
Adiponectin, μg/mL	1.07	1.01–1.13	0.03	1.06	1.01–1.12	0.02

OR: Odds-Ratio; 95% CI: 95% Confidential Intervals; BMI: Body Mass Index. The dependent variable was the presence of proteinuria (>0.5 g/day). Covariates included in this analysis were those variables that had a *p*-value <0.20 in the univariate analysis or those considered with biological plausibility for the development of proteinuria.

For the stepwise method was used forward conditional approach.

## Discussion

We observed that higher serum adiponectin levels were associated with proteinuria in LN patients. Adiponectin levels correlated significantly with the severity of proteinuria in 24 h. After adjusting for confounders in the multivariate analysis, increase in adiponectin levels remains associated with the intensity of proteinuria. On the other hand, serum leptin levels were not related to the presence or severity of proteinuria. These findings are interesting because leptin is associated with the pro-inflammatory properties in some rheumatic autoimmune disorders such as RA [14,29], but their role in SLE patients has not been proved consistently. Instead, adiponectin has a more complex role in rheumatic disorders, and in this study, we established a consistent association between higher adiponectin levels and proteinuria in SLE patients, pointing that this adipokine should be considered as a biomarker of renal involvement in SLE patients. Quantification of the severity of proteinuria constitutes a major clinical biomarker in LN, one of the main parameters for surveillance of renal flare and renal function prognosis [30,31]. Koo et al., in a longitudinal study, observed that remission of proteinuria, defined as a urine protein to creatinine ratio <0.3 during follow-up in LN, can be utilized as a good prognostic indicator for the maintenance of kidney function and long-term survival [32]. Over the last few years, there has been a growing interest in investigating the relevance of adipokines in SLE patients, since they may play a role as immunomodulators of adipocyte-derived cytokines [33]. Leptin participates in the enhancement of some pro-inflammatory responses [34]. Leptin is one of the main adipokines with stronger evidence on rheumatic autoimmune diseases. Abella et al, in an elegant review, summarizes the role of leptin in inflammation and autoimmune disorders [29]. Leptin participates on the modulation of innate and adaptive immunity. On innate immunity, leptin enhances the proliferation and activation of monocyte cells and inhibits the function of natural killer cells whereas on adaptive immunity leptin participates increasing the T and B-cells proliferation, and enhancing the production of pro-inflammatory cytokines [29]. On human chondrocytes, this adipokine increases IL-8 levels and vascular cell adhesion protein 1 (VCAM-1). Induction of this adhesion cell molecule is mediated by JAK2 and P13K [35]. Leptin has pro-inflammatory properties also on other cells increasing IL-6 and IL-8 production by fibroblasts [36]. Adiponectin the second adipokine most evaluated in rheumatic diseases possesses a complex role with some anti-inflammatory effects as well as inhibition of TNF-α and IL-6 production and the induction of IL-10 synthesis[34,37,38]. However, adiponectin may have inflammatory effects, inducing pro-inflammatory chemokines and cytokines production, including IL-8 and monocyte chemoattractant protein -1 (MCP-1) [39]. In vitro studies using cultures of osteoblast, cells adiponectin induces the production of vascular endothelial growth (VEGF), MMP-1, MMP13, IL-6 and IL-8 [35]. Studies on synovial cells derived from RA, adiponectin acts in combination with IL-1β as an inductor of IL-6, IL-8 and PGE2 [8]. This duality of adiponectin behavior has also been observed in chronic inflammatory autoimmune diseases. Choi et al., in patients with RA, reported that adiponectin promotes an inflammatory process and destruction of the joints through the stimulation of vascular endothelial growth factor and matrix metalloproteinases (MMPs) production [40]. Also in RA patients, Frommer et al., observed that adiponectin boosts the synthesis of TNF-α, IL-6, IL-8, and chemokines [41]. There is some information about the relationship between leptin and adiponectin on other renal diseases. In a study carried out by Hayakawa et al., [20] in chronic glomerulonephritis (GN), a positive correlation was observed between plasma adiponectin and proteinuria, and a negative correlation was observed between estimated glomerular filtration rate and plasma adiponectin; nevertheless, they did not find a correlation between leptin with proteinuria in GN [20]. However, this study lacked a control group and did not include SLE patients. In our study, we provided a comparison of leptin and adiponectin concentrations between SLE patients and controls, demonstrating that higher adiponectin levels and an increased in adiponectin/BMI ratio are observed in SLE patients independently of the effect of BMI. With regard to SLE, there are some studies evaluating both adiponectin and leptin, with inconsistent results. Similar to our observations, other authors had found an increased on leptin and adiponectin levels in SLE patients [42,43].

However, there is only limited information on the relationship between these adipokines and kidney involvement in SLE. Our results regarding higher adiponectin levels in LN are supported by Rovin et al., who described that SLE with renal flare had higher plasma adiponectin levels compared with those without renal flare, but they did not examine the presence of leptin [18]. However, Rovin et al., [18], were unable to find any association between plasma adiponectin with clinical parameters of renal flare in their multivariate analysis. Similarly, Loghman et al., [22] observed an increase in urinary adiponectin in patients with LN compared to SLE patients without nephritis. Hutcheson et al., [12] observed increased serum adiponectin and leptin levels in a sample of patients with LN compared with healthy controls. However, these authors were unable to find a correlation between serum leptin and adiponectin with markers of renal damage, and there was no difference between urinary adiponectin in both groups. Different to our findings, Vadacca et al., [11] did not observe differences in serum adiponectin levels between patients with SLE and controls. Unfortunately, in that study, there was no information about adiponectin levels in renal involvement.

Our observations that increases in adiponectin levels are associated with proteinuria in LN patients are supported by studies performed on other diseases. Rovin et al., and Zoccali et al., in independent studies, observed that patients with nephrotic syndrome and end-stage renal disease have higher levels of adiponectin [18,44].

We observed that anti-dsDNA titers were correlated with the severity of proteinuria in LN, and a trend toward antinucleosome antibodies titers and an increase in proteinuria were also noted, although this correlation did not reach statistical significance. Interestingly, these titers of anti-dsDNA or antinucleosome antibodies did not correlate with serum leptin or adiponectin levels, indicating that antinucleosome and anti-dsDNA antibodies are independent markers of adiponectin and probably are a reflex of independent pathways. We observed a positive correlation between adiponectin and the SLEDAI score, despite other studies did not find an association with disease activity in patients with SLE [10,42]. Again, further longitudinal studies should corroborate if high levels of adiponectin are good markers for specific kidney involvement but not for the involvement of other organs, as is observed in this study.

Our study was limited because all patients with SLE were currently receiving treatment with corticosteroids, and high doses of these drugs may increase the serum levels of these adipokines [11,45]. A subsequent study with a subgroup of patients recently diagnosed been naïve to treatment with immunosuppressive drugs or corticosteroid treatment would be useful to exclude the potential confounder of these drugs on adipokines levels. Nevertheless, prednisone doses do not explain the observed association between adiponectin and proteinuria because in the adjusted analysis for potential confounders, adiponectin levels remain associated with proteinuria independently of this confounder. These results highlight the importance of new evaluations of the role of these adipokines, mainly adiponectin, in longitudinal studies.

## Conclusions

Adiponectin concentrations in serum are substantially increased in women with SLE presenting proteinuria in comparison with SLE patients without kidney involvement. In addition, the severity of proteinuria was correlated with an increase in serum adiponectin levels. Higher serum adiponectin levels can be considered as biomarkers for proteinuria in LN independently of other confounders. Longitudinal studies should evaluate whether SLE patients with kidney involvement and high serum adiponectin levels develop different responses to pharmacological treatments for nephritis. Also, additional follow-up studies are required to identify if patients with high adiponectin levels might develop differences in the future probability of impairment in renal function or higher rates of renal relapse.

## Supporting information

S1 Dataset Serum levels of adiponectin and leptin as biomarkers of proteinuria in lupus nephritis dataset. (XLSX)
